# New Insights Into the Peculiar World of the Shepherd-Dog Parasites: An Overview From Maremma (Tuscany, Italy)

**DOI:** 10.3389/fvets.2020.564164

**Published:** 2020-09-25

**Authors:** Benedetto Morandi, Angelica Mazzone, Francesca Gori, Cristian A. Alvarez Rojas, Roberta Galuppi, Peter Deplazes, Giovanni Poglayen

**Affiliations:** ^1^Department of Veterinary Medical Sciences, Alma Mater Studiorum-University of Bologna, Bologna, Italy; ^2^Institute of Parasitology, University of Zurich, Zurich, Switzerland

**Keywords:** shepherd-dog, parasites, taeniids, *E. granulosus*, epidemiology, public health

## Abstract

Several developments have been recently achieved to understand pet-dog parasites and their relationship with hosts; however, parasites' presence and distribution in shepherd-dog have been mainly neglected; this knowledge gap is of critical sanitary importance, as shepherd-dogs could harbor zoonotic helminths including *Echinococcus granulosus sensu lato*. The related human disease, cystic echinococcosis, is a worldwide neglected disease, with high endemicity in the Mediterranean Basin. To evaluate the presence of *E. granulosus* and other parasites, a sheep-dog population from the province of Grosseto (Tuscany, Italy) has been investigated. Overall, 648 dog fecal samples obtained from 50 modern sheep farms, having a total of 216 dogs, were collected. Specimens were analyzed using a standardized centrifugal flotation method (specific gravity = 1.3). Taeniid eggs detected were further isolated using a sieving/flotation technique. DNA was isolated from eggs for PCR and sequence analyses for species identification (gene target: 12S rRNA and *nad1*). Thirty-nine (78%) farms tested positive for at least one parasite species or genus. The most represented intestinal helminths were *Toxocara* spp. in 64% of farms, followed by Ancylostomatidae (58%), *Trichuris vulpis* (50%), *Capillaria* spp. (34%), and taeniids (32%). Sequence analyses confirmed the presence of *Taenia hydatigena* in seven farms, *Taenia* (syn. *Multiceps*) *multiceps* in five farms, and *T. pisiformis* in one farm. No DNA was extracted from four previously taeniid egg-positive farms. No amplification of amplicon corresponding to *E. granulosus* was achieved in the investigated farms. Although not entirely expected, Spearman's test showed a positive correlation between flock size and the number of dogs per farm (ρ = 0.588, *P* < 0.001). The quantitative analysis reported that the home slaughter practice was affected neither by the flock size nor by the number of dogs per farm. The probability to diagnose farms positive for taeniids had been increased by about 35% for each dog unit increase [odds ratio (OR) = 1.35, *P* = 0.012]. In conclusion, the wide distribution of *T. hydatigena* and *T. multiceps* detected in the present study clearly reveals that dogs have still access to raw offal, a major risk for the transmission of *E. granulosus*. Home slaughtering is an unavoidable practice, and more efforts must be undertaken by the public health system to prevent and control potential zoonotic taeniids.

## Introduction

Although progress has been recently made to increase scientific knowledge of pet-dog parasites (1), the same has not been done for shepherd-dog helminths. The incommunicability between pastoralist world and public health system makes the picture even hazier. This is primarily due to the different interests involving the two categories (2). This knowledge gap is of critical sanitary importance, as shepherd-dogs could harbor potentially zoonotic parasites, such as *Echinococcus granulosus sensu lato, Taenia* spp., *Taenia* (syn. *Multiceps*) *multiceps*, and *Taenia serialis*, whose life cycles include the dog as the definitive host and the sheep or other herbivorous as the intermediate host (3, 4). Additionally, cystic echinococcosis (CE), caused by intermediate larval stages of *E. granulosus*, is among the five most frequently diagnosed zoonosis in the Mediterranean Basin (5) and distributed worldwide (6). CE appears differently distributed across the Italian peninsula, showing a hyper-endemic diffusion in the south and being considered sporadic in the north (7, 8). This should not be surprising since sheep domestication started around the fifth century B.C. in the Fertile Crescent (9), and dog breeding for guard and hunting intents started around 15,000 B.P. (10). Since then, dogs and sheep have maintained a strong connection at farm level, sharing parasites. In this context, shepherds play a crucial role in the spread of metacestodoses between sheep and dog by feeding dogs with raw sheep meat and offal, which have been directly slaughtered and butchered on the farm. For example, Singh et al. (11) report that around 60% of the interviewed farmers from New Zealand fed dogs by using home-slaughtered “meat.”

Diagnosis and detection of *E. granulosus* into the definitive host are key points in developing epidemiological studies and implementing hydatid control programs in endemic areas (12). Generally, two paths are available to detect taeniids from the small intestine: *ante-mortem* and *post-mortem* examinations. Obviously, the latter is not always possible, although necropsy has shown 100% specificity and 97% sensitivity, even at a very low parasite burdens (<6 worms) (12) and remains the gold standard for the detection of adult tapeworms (13). However, both may impose a risk to public health; therefore, appropriate measures must be taken to reduce the zoonotic impact (13). *In-vitam* examination is performed through several laboratory techniques using different matrices, such as sera and feces. Indirect diagnosis, such as ELISA tests performed on serum, have been attempted showing variable sensitivities, ranging from 40 to 90% (14); however, they are not routinely used. Tests for the detection of *Echinococcus* coproantigens based on ELISAs have been developed by several research groups (12, 15). These tests have been used mainly in control programs, although some cross-reactions with other intestinal cestodes have been observed (16). On the other hand, molecular analysis of feces showed very low sensitivity, as it yields 74% false-negative results when performed from 21 to 31 days post-infection (17). Furthermore, copro-PCR is challenging even after 31 days post-infection, as DNA extraction from fecal samples is complicated by the presence of inhibitory substances (12).

All the tests mentioned above share the feature of being useful for monospecific parasite detection (18). Parasite concentration by coprological flotation is a classical approach for a variety of intestinal parasites, with variable specificity and sensitivity restricted to the patent period only (19). Parasite stages excreted with feces can be classically differentiated by the morphology of eggs, cysts, or oocysts and more precisely by morphometry (e.g., *Toxocara* spp., hookworms, *Capillaria* spp., and oocysts) (20).

Taeniid eggs, which are discontinuously shed, cannot be differentiated by light microscopy (21).

The detection of the eggs in fecal samples after concentration by traditional routine diagnostic methods is claimed to suffer from low sensitivity (21); however, so far, it has not been evaluated for all taeniid species. The enrichment of taeniid eggs and their subsequent genetic analysis can overcome this limitation and open new diagnostic strategies. Efficient enrichment of taeniid eggs was achieved by a combination of sequential sieving and flotation in zinc chloride solution (F/Si method) (22). In a field study in Lithuania, significantly more dogs excreting taeniid eggs were diagnosed by the F/Si method (34 of 240 dogs investigated) as compared with 12 positive animals identified with the modified McMaster method, an approach known to have low sensitivity. Genetic analyses performed on the 34 egg sediments identified by the F/Si method revealed nine *E. granulosus s.l*. and two *Echinococcus multilocularis* infections, but only one of these *Echinococcus*-positive animals was identified when using the McMaster method as a screening test (23), documenting, that in general lower eggs per gram (EPGs) are present in *Echinococcus* as compared with *Taenia* infections. However, other screening methods for the isolation of taeniid eggs were more sensitive and comparable with the F/Si method (i.e., flotation Ovassay technique) (24–26).

Following egg isolation with any of the aforementioned methods, genetic analyses with specific primers can be performed [primers for *Echinococcus* spp.; see (12, 18)]. A poly-specific approach based on targets in mitochondrial genes with a multiplex PCR allows the differentiation among *E. multilocularis, E. granulosus s.l*. other cestodes from canines (27). Sequence analyses of the amplicons for “other cestodes” allow further identification of some *Taenia* spp. (*Taenia hydatigena, Taenia ovis, Taenia taeniaeformis, Taenia polyacantha, Taenia pisiformis*, and *Taenia crassiceps* but cannot clearly differentiate between *T. multiceps* and *Taenia krabbei* with the currently available molecular data). Identification of *Taenia* to the species level is of value in *Echinococcus* control programs or in very low endemic areas to trace back *Taenia* infections in dogs, typically originating after ingestion of infected farm animals (*T. hydatigena, T. ovis*, and *T. multiceps*) or from rodent and lagomorph intermediate hosts (*T. crassiceps, T. polyacantha, T. taeniaeformis*, and *T. serialis*) (18).

Furthermore, this poly-specific approach has successfully been used in investigations of wild carnivores, in foxes (18), or wolves in Italy (28) and Portugal (29) documenting their involvement in taeniid cycles.

Due to the lack of available data, this field study aims to contribute to the knowledge about the frequency of shepherd-dog parasites at farm level, mainly focusing on *E. granulosus* in an endemic area in Tuscany region, Central Italy (7).

## Materials and Methods

### Study Area

**Figure 1:**
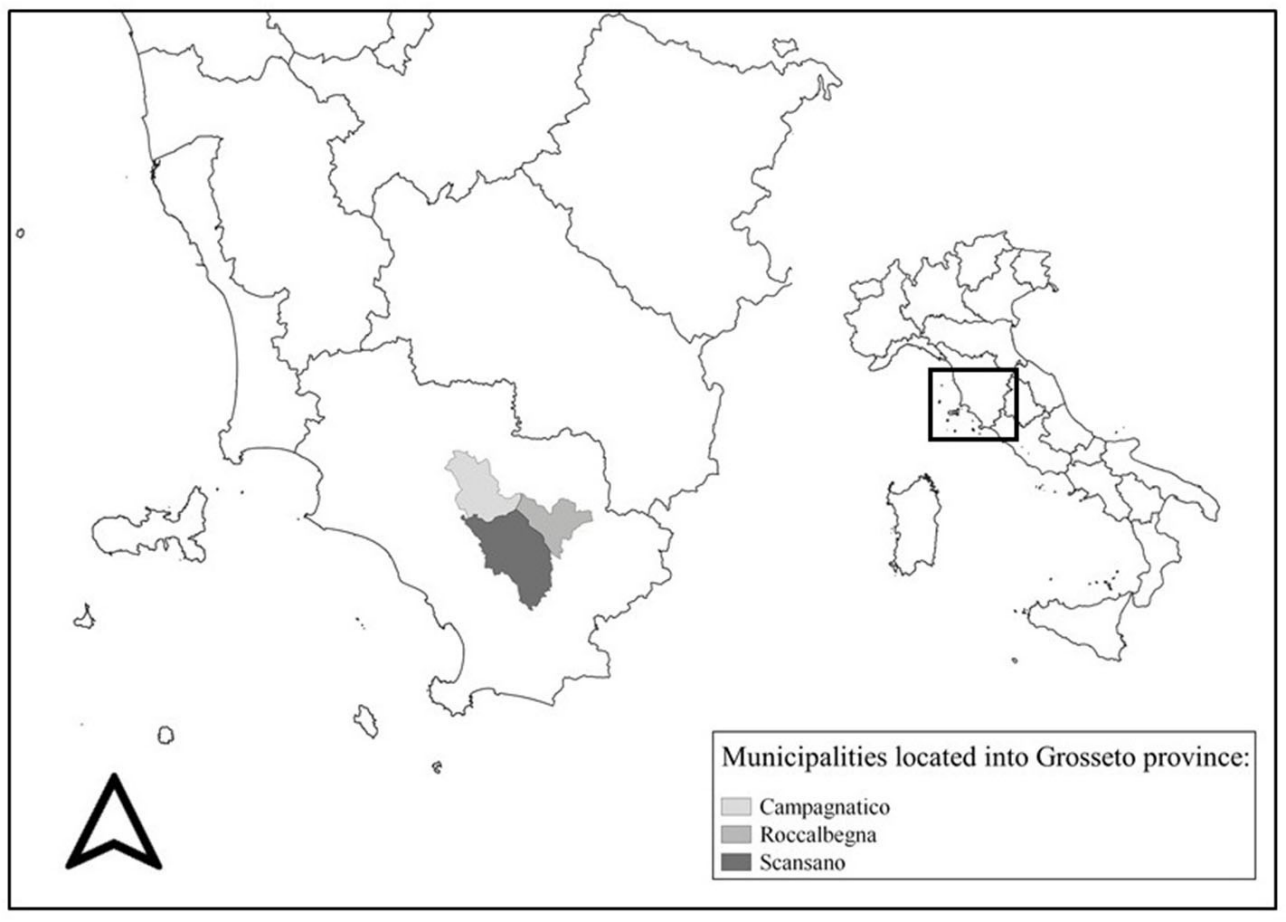
Gray scale represents the three municipalities where the involved farms were located.

The activity was performed in the Southern area of Tuscany (province of Grosseto, Central Italy), named Maremma (from Latin *maritima*, “maritime”) (Figure 1). This subregion is suited to animal breeding, namely, sheep, cattle, and horses. The area extends for about 5,000 km^2^, covering two regions and five provinces. The hilly municipalities of Campagnatico, Roccalbegna, and Scansano, within the Grosseto province, consist of an area of nearly 56,000 ha, where a total of 46,238 sheep heads over 203 breeding units are farmed. The 203 registered farms in the area are distributed as follows: 51 with 14,920 heads in Campagnatico's municipality; 38 in Roccalbegna hosting 7,784 heads, and 114 in Scansano with 23,534 heads.

### Inclusion Criteria and Fecal Sampling

A preliminary list of the total number of the farms was provided by Public Health Services veterinarians and workers of CIA (Confederazione Italiana Agricoltori). Firstly, farms not having dogs were excluded from the study. Furthermore, only farms with at least one dog, able to either have contact with the sheep flock or accede to the pastures, were included. Finally, also based on the willingness of the farmers to be involved, a subset of 50 farms remained in the study.

The selected farms were visited between May 2016 and February 2017. Animal-level and farm-level data were collected during each visit. Farmers were asked to answer a questionnaire, and data were entered into MS Excel (Microsoft Inc., Sacramento, California, USA). Animal-level information included the number and the species of farmed animals and the number of dogs in the farms. Farm-level information included the sheep production system (meat, dairy, or both), prophylactic measures against infectious diseases, and GPS coordinates. Data reporting home slaughter practices were also obtained during the visit.

Randomly walking throughout the property at the time of the visit, three dog fecal samples, detected directly on the ground, were picked up per dog present on the farm. Each fecal sample was labeled and stored into a plastic bag individually and subsequently placed into a refrigerated container/bag. As a biosafety precaution, samples were stored for 10 days at −80°C (30) and then at −20°C until examination.

### Parasite Collection

Up to 5 g of feces for each sample was analyzed. Parasite elements were concentrated from fecal specimens by using the Di Felice and Ferretti (31) solution (sodium nitrate and sugar; specific gravity = 1.3) as flotation media in a standardized centrifugal flotation method (32). When positive for taeniid eggs, the corresponding leftover samples were stored at −20°C for further egg isolation/PCR aimed at achieving species identification.

### Genetic Identification of Taeniid Eggs

Taeniid eggs were isolated with a combination of flotation in zinc chloride solution followed by sequential sieving (F/Si method) (22). DNA extraction was carried out following Štefanić et al. (33), and species identification of taeniid egg was performed using a multiplex PCR, according to Trachsel et al. (27), using a Qiagen multiplex PCR kit (Qiagen, Hilden, Germany). Furthermore, “other cestode” amplicons were sequenced, after purification using the MinElute PCR purification kit (Qiagen, Hilden, Germany) according to the manufacturer's instructions. Sequencing was performed by Microsynth, Switzerland. Sequencing results were compared with reference mitochondrial genes for all taeniid species retrieved from GenBank.

### Statistical Analysis

Data collected through in-person questionnaires were merged with laboratory results into a MS Excel spreadsheet and then imported to Stata 15 (StataCorp LLC, College Station, Texas, USA) for analyses. The number of sheep and dogs was interpreted as independent variables and correlated to the home slaughter practice and the presence/absence of taeniids into the farms. Continuous data that were non-normally distributed were summarized using medians and inter-quartile ranges (IQRs), while normally distributed data were summarized using mean ± *SD*. Spearman's correlation test was computed to assess the relationship between sheep and dogs, as their association is not always, at least in Italy, rational and predictable. Fisher's exact test was applied, as more than 20% of cells had expected frequencies < 5, to compare groups (34). When reasonable, odds ratios (OR) and relative 95% confidence intervals (CIs) have been assessed as measures of association to explore the effect of independent over dependent variables. Results were considered significant when *P* ≤ 0.05.

## Results

**Figure 2:**
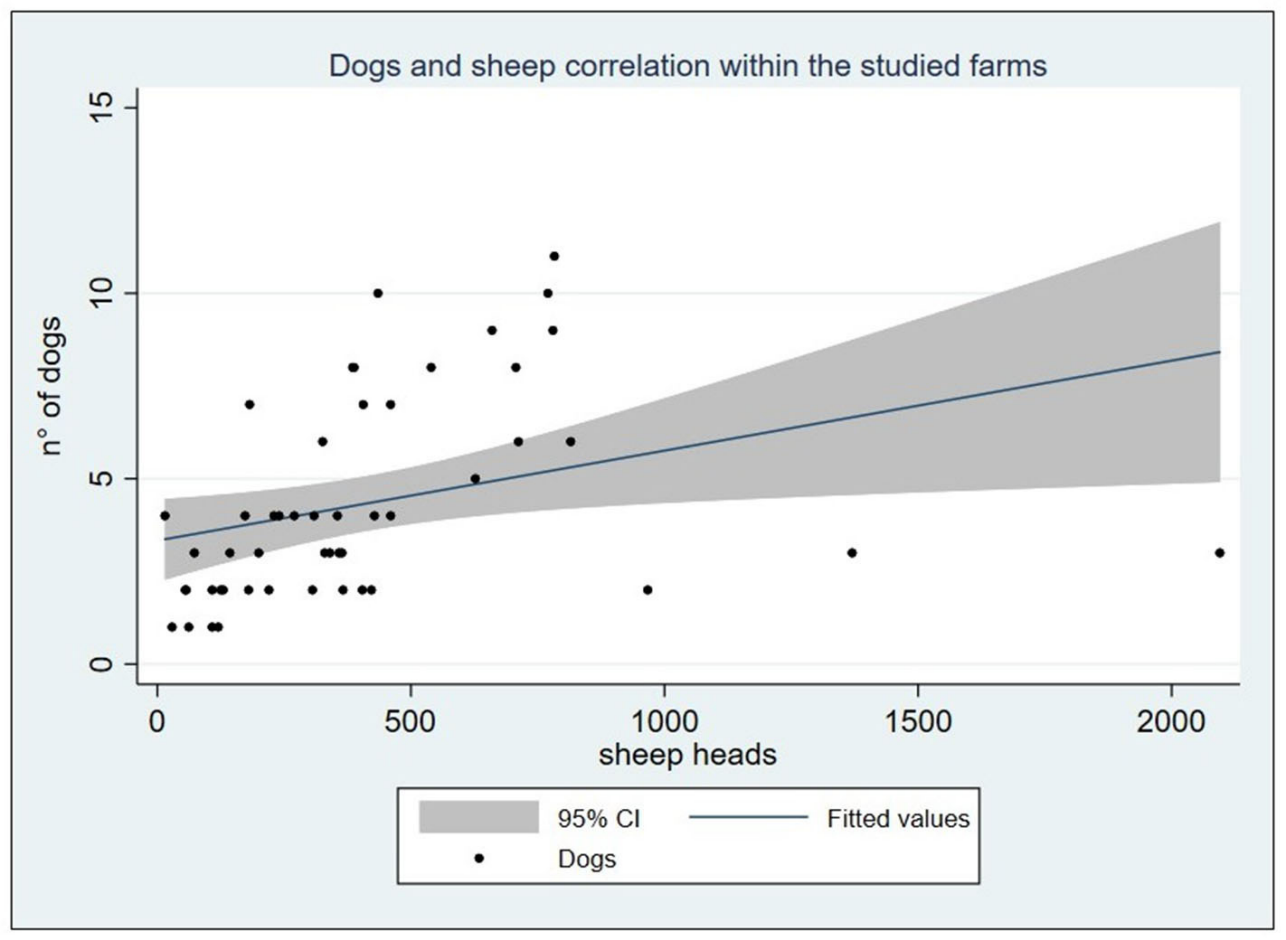
Graph reporting the positive linear correlation between the sheep and dog count within the studied farms (ρ = 0.588, *P* < 0.0001).

A total of 50 sheep farms were visited from May 2016 to February 2017. Overall, farms hosted 20,388 sheep with a median of 347.5 ranging from 15 to 2,095 heads (IQR: 173–460). Dogs, including livestock guarding dogs, shepherd-dogs, hunting dogs, and pets, accounted for a total of 216 (Table 1 shows dogs' frequency by farm), showing a mean of 4.3 ± 2.72 with a range of 1–11. Considering the livestock guarding dogs and shepherd-dog categories, the average dog/sheep ratio was 1:114.5. Farms with one dog had a mean of 79.75 (*SD* ± 42.1) sheep, while the only farm with 11 dogs had 783 heads. Spearman's correlation test highlighted that the number of dogs hosted in the farms was positively correlated to the number of sheep (ρ = 0.588, *P* < 0.0001), as shown in Figure 2. Farms included in the study were mostly dairy farms (70%), while 10 were specialized in the production of sheep meat, and only three had both. Home slaughter appeared to be widely spread among farms, since 40 out of 50 farmers declared to practice it. No statistically significant differences emerged comparing the different production systems and the home slaughter practice (Fisher's exact χ^2^ = 3.581, *P* = 0.26).

**Table 1:** Number of farms with the specific number of dogs, their frequency, and the relative collected samples.

**No. of dogs *per* farm**	**No. of farms**	**Freq %**	**No. of collected samples**
1	4	8	3
2	12	24	6
3	9	18	9
4	9	18	12
5	1	2	15
6	3	6	18
7	3	6	21
8	4	8	24
9	2	4	27
10	2	4	30
11	1	2	33

Concerning dog prophylaxis procedures, only 19 farmers out of 50 (38%) reported the administration of anthelmintic drugs to their dogs and stated that they either did not remember the used products or had administered off-label avermectins. None of the farmers mentioned the use of praziquantel, which is the appropriate treatment for taeniids.

**Figure 3:**
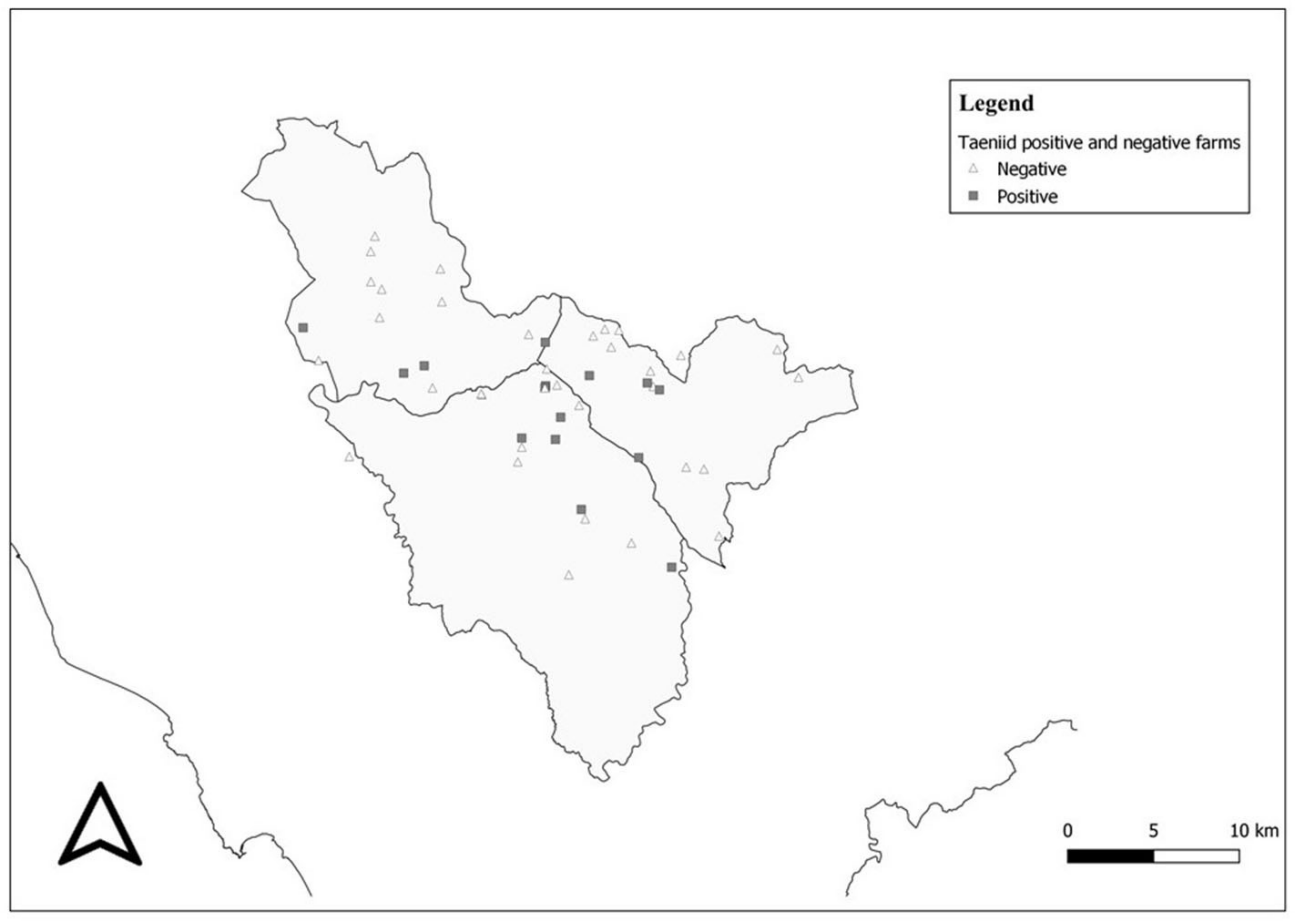
Taeniid-positive and taeniid-negative farms distributed within the study area.

A total of 648 fecal samples, based on 216 dogs present into the 50 visited farms, were processed and investigated for the presence of parasites. The crude coprological results revealed a high proportion of positivity for parasites at the farm level since 78% (39/50) were positive for at least one parasite (Figure 3). Out of 648 samples analyzed, 312 showed parasitic elements resulting in a 48.1% frequency. Table 2 shows the five groups of parasites diagnosed by using flotation technique. Specimens with multiple infections were slightly less common than the single infection, with 54.8 and 45.2%, respectively. On the contrary, farms having multiple parasite species were far more common than those with single-parasite type, with 89.7 and 10.3%, respectively (Table 3).

**Table 2:** Reported frequencies of detected parasites in the surveyed farms.

**Recovered parasites at farm level**	**No. of positive farms**	**Frequency%**
*Toxocara* spp.	32	64
Ancylostomatidae	29	58
*Trichuris vulpis*	25	50
*Capillaria* spp.	17	34
Taeniids	16	32

**Table 3:** Proportions of single and multiple parasite species detected in the 39 positive farms and in the 312 fecal positive-diagnosed samples.

**No. of different parasitism**	**Farm level**		**Fecal samples**	
	***n***	**%**	***n***	**%**
1	4	10.3	171	54.8
2	8	20.5	100	32.1
3	14	35.9	34	10.9
4	8	20.5	6	1.9
5	5	12.8	1	0.3
Total	39	100	312	100

A total of 47 fecal samples from 16 different farms (farm-level frequency: 32%) revealed taeniid eggs. PCR product after multiplex PCR for taeniids was acquired in 34 out of 47 positive samples. In terms of frequency among positive samples for taeniids, *Taenia hydatigena* was the most common isolated species (31.9%), followed by *Taenia* (syn. *Multiceps*) *multiceps* (21.3%) and *Taenia pisiformis* (2.1%). Electropherograms from eight sequences were not of high quality or were too short to be able to identify the species; then they were identified as *Taenia* spp. No sample was positive for *Echinococcus multilocularis* and/or *E. granulosus s.l*. As regards taeniids' frequency at farm level, *T. hydatigena* was the most commonly detected species at 43.7% (seven out of 16 taeniid positive farms), followed by *T. multiceps* 31.2%; and one farm was positive for *T. pisiformis*. No PCR product was obtained in samples from four different farms.

Three farms were detected having multiple taeniids infections, particularly one with *T. hydatigena, T. multiceps*, and *Taenia* spp.; one with *T. multiceps* and *Taenia* spp.; and finally one with *T. multiceps* and *Taenia* spp. No taeniid eggs were detected in home slaughter-free farms (10 out of 50). Twenty-seven farmers reported to have knowledge of CE; eight of them stated to have seen at least one hydatid cyst.

Quantitative analysis, carried out by estimating the ORs, suggested that home slaughter practice was not affected by the flock size. Similarly, the number of dogs did not increase the probability to perform a home slaughtering. On the other hand, for each dog unit increase into the farm, the chance of having circulating taeniids increased by a factor of around 35% (OR = 1.35, *P* = 0.012). Quantitative results are summarized in Table 4. Finally, differences in circulating taeniids among different production systems did not show any statistical significance (Fisher's exact = 2.32, *P* = 0.32).

**Table 4:** Farm-level odds ratios, *P*-values, and relative 95% CIs among farmer-reported predictors and outcomes.

**Outcomes**	**Predictors**	**Odds ratios**	***P*-values**	**95% CIs**
Home slaughtering	Flock size	1.001	0.382	0.99–1.003
Home slaughtering	No. of dogs	1.12	0.421	0.84–1.49
Taeniids	No. of dogs	1.35	0.012^*^	1.07–1.72

* *Statistically significant result*.

## Discussion

The present study offered useful information on the distribution of endoparasites, particularly taeniids, in shepherd-dogs from Maremma's (Tuscany region) sheep farms and provided interesting insights on a few practices commonly adopted by farmers.

Farmers participating with the study were breeding 20,388 sheep, representing almost half of the total number of sheep in the three municipalities; furthermore, the 50 farms considered for the study represented around one fourth of the total 203 sheep farms registered in the area. Assuming that the larger the flock size the more advanced the management systems adopted by the farm, the sampled farms were slightly more technologized than the non-responding ones; this aspect might have represented a selection bias, which resulted in an underestimation of parasite frequencies.

Data on the optimal dogs/sheep ratio providing the best benefit in terms of livestock guarding or herding are lacking. On average, the dog/sheep ratio of 1:114.5 was reasonable as recommended by Gemmell et al. (35), who also urged a drastic reduction and control of dog population size, as a pillar in the control of echinococcosis. As regards the studied farms, Spearman's correlation showed a moderate positive association between the number of dogs per farm and the flock size, demonstrating a non-random allocation. Knowledge about the existence of a rational association among flock size and dog units is useful, if not essential, for the control of zoonoses related to shepherd-dogs (35). All the dogs present into the farms were officially registered, and no stray dogs were reported in the study area.

Farmers did not report which drugs were used as a treatment or as prophylaxes for canine parasitic diseases, which is a commonly noticed behavior (36, 37). Farmers tend to treat dogs using products for sheep simultaneously when deworming the flocks; these products are mostly represented by avermectins, which are useless for taeniids. Furthermore, our results highlighted the presence of nematodes and cestodes, pointing out how this approach is both futile and antieconomic. This practice confirms that shepherd-dog parasites are widely neglected and underestimated among breeders. Attention to dog health issues is insufficient, probably because dogs are not considered as a direct source of income, contrary to the sheep flock.

Diagnosed nematodes are commonly reported also from pets (i.e., ascarids, hookworms, and trichurids) (1). Ascarids were the most common nematodes detected on the farms. The different modes of infection transmission and resistance of the eggs in the environment may lead to a cumulative environmental contamination, representing a risk for human infection (38). Hookworms were found in 29 farms; this result is consistent with the prevailing opinion that these parasites are related to rural environments (39). Additionally, the zoonotic potential of hookworms should not be underestimated as they may induce two severe conditions known as human gut disease (eosinophilic enteritis) and cutaneous larva migrans (CLM) or creeping eruption (40). As for *Trichuris vulpis*, its zoonotic potential is still being debated. Cases of visceral larva migrans (VLM), described in the literature, have been reviewed by Traversa (41), even though dog whipworms are generally not reported as zoonotic pet intestinal nematodes (42).

By comparing our results with data from studies regarding feces randomly collected from soil in urbanized areas of Italy that report positivity of always around 17%, we recorded a higher frequency per sample (43, 44). Additionally, a conference abstract of a nationwide study on owned dogs with constant or regular access to the outdoors, carried out in Italy by Brianti et al. (45), reports a much higher overall prevalence of around 30%. When our frequencies are compared with results obtained within a similar environment, percentages are close (46). Nevertheless, an underestimation of the real prevalence is likely in this study. This may be due primarily to the low sensitivity of the flotation technique (47) and secondly to the effect of freezing, which has been reported to mask low-intensity infections (48). As for farm level, parasites were recovered in approximately 80% surveyed farms, with 90% of positive farms showing multiple parasite infections. These data are higher than the data of Phythian et al. (49), who reported 50% positive farms in a survey carried out in South-Western England. This difference might be due to different sampling methods: in the present study, three stools from each dog present in the farm were randomly collected on the ground (see Table 1), whereas Phytian et al. (49) only sampled one stool. Particularly noteworthy is the fact that, as expected, multiple infections were more common at the farm level than at the sample level, where the majority of samples resulted positive for a single-parasite group (Table 3). This could be due to the fact that a farm may have more than one dog mono-parasitized by different parasites.

As often reported, taeniid presence within farms is strictly related to the traditional home slaughtering: indeed, taeniid eggs were not detected in the 10 farms where home slaughter was not reported. Adult sheep meat trade is currently increasing due to the spread of *halal* food market and to typical recipes of the Italian gastronomy, such as sheep skewers, salami, and ham.

Our results highlight how home slaughtering without any veterinary control remains the major risk for the spreading of potentially zoonotic tapeworms. *Taenia hydatigena* was the most represented tapeworm, with around 44% of the farms being positives. A quite recent systematic review focused on the zoonotic potential linked to parasites of carnivores in Iran (50), encompassing studies from 1997 to 2015, reported that *T. hydatigena* was the most frequently isolated parasite in dogs, with a prevalence of around 30% out of 1,539 examined dogs. Usually, *T. hydatigena* represents the most common taeniid species detected worldwide in both domestic (51) and wild environment (28), where the wolf act as the main definitive host (52). This ecological success is probably due to the short period of 5–8 weeks required for the maturation of cysticerci (53).

The second most common tapeworm species identified in this study was *Taenia multiceps*, whose larval stage is a coenurus mainly localized in the central nervous system of small ruminants, which produces a well-known clinical syndrome. *T. multiceps* is worldwide distributed, mainly reported in young animals between 3 and 6 months of age and, accidentally, in adult sheep younger than 18 months (54). Many mammals, including sheep, goats, horses, cattle, camels, deer, and pigs, may serve as intermediate hosts (55). The associated disease in sheep is named “gid” or “sturdy” and has an acute or chronic phase. Due to the shorter prepatent period compared to *E. granulosus*, both clinical presentations might be a warning against the habit to give raw offal to dogs. Additionally, *T. multiceps* coenurosis is a zoonotic infection with more than 50 human cases described in the literature (56), several of which have also been reported in Italy (57), including five from Sardinia (58).

The single sample positive to *Taenia pisiformis* in a farm, where domestic rabbits were absent, suggests the administration of hunting offal.

As expected, none of the samples were positive for *Echinococcus multilocularis*, which has never been reported from the area; surprisingly, *E. granulosus* (sheep-strain G1) was not detected either. Sardinia, where a hyper-endemic scenario for *E. granulosus* is present, reports the highest frequencies around 10%, depending on which ELISA test was utilized (36). Since *E. granulosus* is considered diffused all over Italy (8), its absence in the present study could be linked to the positive feedback of a specific educational course offered by Public Veterinary Services to farmers a few years earlier (as reported by farmers during the visits). However, the wide presence of *T. hydatigena* and *T. multiceps* clearly shows that dogs have still access to raw offal, a major risk for the transmission of *E. granulosus*. These data might also suggest that farmers have learned to recognize and discard only hydatid cysts. Unfortunately, no target DNA was amplified from 14 previously positive samples, possibly due to the low burden of infection. Likewise, in eight samples, it was not possible to identify the *Taenia* species responsible for infection, possibly due to an insufficient amount of DNA or the occurrence of a double infection.

All over Italy, the economic value of an old sheep (higher risk category for the presence of cysts) is very low, and it almost forces farmers to practice home slaughtering. According to our survey, the home slaughter practice was almost equally performed in different size farms, and it was not statistically dependent on the flock size. On the contrary, the prevalence of tapeworms was highly related to the number of dogs, as the probability to have taeniids into a farm had a 35% increase for each dog unit growth. Therefore, a rationalization of the number of dogs in the farm would be desirable in order to control tapeworm infection. Despite that swine home slaughter is subjected to veterinary inspection (regulated by law DL 333/98) (59), small ruminant home slaughter still requires a specific regulation, being a major risk control point for zoonotic parasitic diseases.

## Conclusion

The present descriptive study highlights the importance of shepherd-dog parasites for public health. Data reporting working-dog parasites are lacking in the scientific literature, and the few published papers are specifically focused on *E. granulosus*. The homogeneity of the area, its own pastoralist vocation, and the high amount (648) of dog fecal samples analyzed from 50 farms offer a reliable picture of the area. The diagnosis of potentially zoonotic helminths as *Taenia multiceps* and *Toxocara* spp. should not be underestimated; and a health care of shepherd-dogs, following ESCCAP guidelines, is needed. Although this study has been carried out in an area where the pastoralism is traditionally advanced, the higher frequency of parasitism in shepherd-dogs compared with companion ones (1) does prove insufficient attention towardz dog health and welfare issues and suggests a lack of veterinary support. Indeed, regardless of the parasite involved, the occasional treatments in dogs, using sheep drugs, do not show real effectiveness. Small ruminant breeding has represented an important economic pillar in the whole Mediterranean Basin for ages; thus, it is time that public and private veterinarians cooperate for a pastoral upgrading all over the entire context.

## Data Availability Statement

The raw data supporting the conclusions of this article will be made available by the authors, without undue reservation.

## Ethics Statement

The participants provided their written informed consent to participate in this study.

## Author Contributions

BM, AM, RG, and GP have thought the project and collected and analyzed the samples. PD, CA, and FG have performed the molecular analyses. All Authors have written and revised the manuscript.

## Conflict of Interest

The authors declare that the research was conducted in the absence of any commercial or financial relationships that could be construed as a potential conflict of interest.
